# *Trypanosoma cruzi* Discret Typing Units (TcII and TcVI) in samples of patients from two municipalities of the Jequitinhonha Valley, MG, Brazil, using two molecular typing strategies

**DOI:** 10.1186/s13071-015-1161-2

**Published:** 2015-10-31

**Authors:** Maykon Tavares de Oliveira, Girley Francisco Machado de Assis, Jaquelline Carla Valamiel Oliveira e Silva, Evandro Marques Menezes Machado, Glenda Nicioli da Silva, Vanja Maria Veloso, Andrea Mara Macedo, Helen Rodrigues Martins, Marta de Lana

**Affiliations:** Núcleo de Pesquisas em Ciências Biológicas (NUPEB), Universidade Federal de Ouro Preto (UFOP), Campus Universitário Morro do Cruzeiro, CEP: 35400-000, Ouro Preto, MG Brazil; Departamento- Básico de Saúde, Universidade Federal de Juiz de Fora (UFJF), CEP: 35010-177, Campus Governador Valadares, Governador Valadares, MG Brazil; Departamento de Análises Clínicas, Escola de Farmácia, UFOP, CEP: 35400-000 Campus Universitário Morro do Cruzeiro, CEP: 35400-000, Ouro Preto, MG Brazil; Programa de Pós-Graduação em Ciências Farmacêuticas (CiPHARMA), Escola de Farmácia, UFOP, Campus Universitário Morro do Cruzeiro, 35400-000, Ouro Preto, MG Brazil; Departamento de Farmácia, Faculdade de Ciências Biológicas e da Saúde, Universidade dos Vales do Jequitinhonha e Mucuri (UFVJM), 39100-000 Diamantina, MG Brazil; Departamento de Bioquímica e Imunologia, Instituto de Ciências Biológicas (ICB), Universidade Federal de Minas Gerais (UFMG), 6627, Belo Horizonte, 31270-901 MG Brazil

**Keywords:** *Trypanosoma cruzi*, Genotyping, *T. cruzi* DTUs, Chronic patients, Jequitinhonha Valley, MG, Brazil

## Abstract

**Background:**

*Trypanosoma cruzi* is classified into six discrete taxonomic units (DTUs). For this classification, different biological markers and classification criteria have been used. The objective was to identify the genetic profile of *T. cruzi* samples isolated from patients of two municipalities of Jequitinhonha Valley, MG, Brazil.

**Methods:**

Molecular characterization was performed using two different criteria for *T. cruzi* typing to characterize 63 *T. cruzi* samples isolated from chronic Chagas disease patients. The characterizations followed two distinct methodologies. Additionally, the RAPD technique was used to evaluate the existence of genetic intragroup variability.

**Results:**

The first methodology identified 89 % of the samples as TcII, but it was not possible to define the genetic identity of seven isolates. The results obtained with the second methodology corroborated the classification as TcII of the same samples and defined the classification of the other seven as TcVI. RAPD analysis showed lower intra-group variability in TcII.

**Conclusions:**

The results confirmed the preliminary data obtained in other municipalities of the Jequitinhonha Valley, showing a predominance of TcII, similar to that verified in northeast/south axis of Brazil and the first detection of TcVI in the study region. The second protocol was more simple and reliable to identify samples of hybrid character.

## Background

At present approximately 6 to 7 million people are estimated to be infected worldwide with *Trypanosoma cruzi,* the etiologic agent of Chagas disease, mostly in Latin America where Chagas disease is endemic [1]. *T. cruzi,* is a flagellate digenetic protozoan belonging to the order Kinetoplastida, family Trypanosomatidae [2]*,* dispersed throughout the American continent from Argentina and Chile to the southern United States of America. Several studies have demonstrated that this protozoan is heterogeneous, consisting of several sub-populations of parasites that circulate in both, domestic and wild environments, with a high rate of biological and genetic diversity [3–5].

Currently, according to the second taxonomic consensus for *T. cruzi* approved during the XXV Protozoology Meeting held in Buzios, RJ, Brazil, the species is subdivided into six discrete typing units (DTU) named TcI, TcII, TcIII, TcIV, TcV and TcVI [6], related to several previous classifications based on different molecular markers. Regarding the geographical distribution of the *T. cruzi* genotypes, it has been demonstrated that TcI has the largest distribution in all America. In Colombia, Mexico, Guatemala, Venezuela, Panama and Bolivia there is evidence of a predominance of this DTU circulating in the sylvatic [7] and domestic cycles, associated in some cases to cardiac clinical forms in humans [8–10]. In the Southern Cone countries, both DTUs (TcI and TcII) were observed in the sylvatic cycle [8–10]. However, only TcII was predominantly associated with human infection, while TcI was rarely found in humans [11, 12]. *T. cruzi* III was detected in human infections [8] and both, TcIII and TcIV [8, 9] are mainly encountered in the sylvatic and domestic cycles. TcII, TcV and TcVI are frequently isolated from infected individuals in the south of America but rarely isolated from sylvatic transmission cycles [13, 14].

Although few studies have been accomplished in Brazil concerning lesser *T. cruzi* subdivisions, there is evidence that the majority of the strains isolated from patients belong to TcII [11, 15, 16] and less frequently to TcV; except in the Amazon Basin where TcI is the most prevalent DTU infecting humans while TcIII and TcIV DTUs were occasionally recorded [17, 18]. At present in Brazil, the TcII strains seem to be more associated with human infections responsible for tissue damage, and consequently with several clinical forms of Chagas disease, while cases of human infections caused by TcI strains are still rare and usually asymptomatic [19], despite the recording of some symptomatic cases of Chagas disease in the Amazon with cardiac manifestations [20, 21].

Due to the scarcity of publications regarding the geographic distribution of the newly classified *T. cruzi* DTUs, including in Brazil, this study proposed to characterize genetically samples of this parasite isolated from patients with chronic Chagas disease living in an important endemic area of Brazil named Jequitinhonha Valley. We aimed to highlight that the knowledge of the distribution and intragroup variability of the newly categorized *T. cruzi* genotypes in the domestic cycle of Chagas disease in this region, where all severe clinical forms of the disease are present, may provide additional contributions to further investigation of the association between the *T. cruzi* genotype and the pathophysiological aspects of this disease, not evaluated yet, continuously researched by several authors [16, 22].

## Methods

### Patients and samples of *T. cruzi*

The samples of *T. cruzi* (n = 63) evaluated in this study were isolated from patients in the early (7/63 patients with less than 14 years old) and later chronic phases (56/63) of Chagas disease, all born and living in the municipalities of Berilo (62 patients) and José Gonçalves de Minas (only one patient), distant 24 km, both of the Jequitinhonha Valley, MG, Brazil. There were 19 male and 44 female patients, aged between 7 and 73 years. For isolation of the parasites the hemoculture technique [23] was used. In addition, the reference clones of the six *T. cruzi* DTUs, kindly provided by Dr. Michel Tibayrenc (IRD, France), were also characterized in parallel: TcI (P209 cl1, 92101601P cl1 and Cutia cl1), TcII (MAS cl1 and Tu18 clI), TcIII (CM-17 and X-109/2), TcIV (CAnIII cl1 and 92122102R), TcV (BUG2148 cl1 and SO3 cl5) and TcVI (P63 cl1 and Tulahuen cl2).

### Preparation of *T. cruzi* cell pellets

After isolation by hemoculture parasites were maintained in growth by successive addition of LIT (Liver Infusion Tryptose) medium up 35 ml of culture. Then, the cultures were subjected to four cycles of washing and centrifugation, using sterile phosphate buffered saline (PBS) at 3500 rpm, 4 °C to prepare damp mass.

### Extraction of DNA

DNA extraction was processed after thawing and homogenization of the wet mass of each *T. cruzi* sample. DNA from the samples was obtained using the WizardTM Genomic DNA Purification Kit (Promega, Madison, WI, USA), following the manufacturer's instructions. For molecular analysis, the DNA samples were diluted at a concentration of 3 ng/μL. Control DNA and reagent-free samples were processed in parallel.

### Criteria for *T. cruzi* genotyping

The criteria [24] and [16] showed in Table 1 were used for *T. cruzi* genotyping, both recommended by the expert committee [4]. In the genotyping protocol [24] the samples were subjected to a PCR algorithm for DTU genotyping which combines the analyses of the polymorphism of the *24sα*-LSU rDNA gene as well as the profile of bands obtained after PCR-RFLP of the *HSP60* and *GPI* genes. In the protocol [16] the *24Sα*-LSU rDNA miniexons and the profile of bands observed after PCR-RFLP of subunit II of the Cytochrome oxidase gene polymorphism were analyzed.

**Table 1:** Genotyping of *Trypanosoma cruzi* isolates into DTUs (TcI – TcVI) according to the methodologies of [24] and [16]

*T. cruzi* DTU	*24sα* rDNA Souto et al. (1996)	RFLP-*HSP60* Sturm et al.(2003)	RFLP-*GPI* Westenberger et al. (2005)	RFLP-*COXII* Freitas et al. (2006)	SL-IR Burgos et al. (2007)
	DNA fragments in base pairs and the number of bands expected				
TcI	110 bp	1 band	2 bands	Haplotype A (262 bp + 81 bp + 30 bp)	~150/157 bp
TcII	125 bp	1 band	3 bands	Haplotype C (212 bp + 81 bp)	~150/157 bp
TcIII	110 bp	2 bands	2 bands	Haplotype B (294 bp + 81 bp)	200 bp
TcIV	~120 bp	1 band	3 bands	Haplotype B (294 bp + 81 bp)	200 bp
TcV	110 bp + 125 bp	3 bands	4 bands	Haplotype B (294 bp + 81 bp)	~150/157 bp
TcVI	125 bp	3 bands	4 bands	Haplotype B (294 bp + 81 bp)	~150/157 bp

### Amplification of the 3' region of the rDNA gene *24Sα*-LSU rDNA

All DNA samples were subjected to three successive PCR amplifications of the divergent domain D7 of *24Sα* subunit rDNA (LSU rDNA) according to methodology [25] using a thermocycler (Biocycler MJ96G). The PCR products were subjected to electrophoresis in 6 % polyacrylamide gel and revealed by silver staining [26].

### PCR-RFLP (restriction fragment length polymorphism) of *HSP60* (heat shock protein) and *GPI* (glucose 6-phosphate isomerase) genes

The polymorphism of HSP60 (heat shock protein) and GPI (glucose 6-phosphate isomerase) genes for the populations of *T. cruzi* was evaluated according to the protocol [27] using the primers *HSP60-1* and *HSP60-2* described in [28] for *HSP60* and the primers SO1 and SO2 described in [29] for GPI.

PCR was performed using the thermocycler Biocycler MJ 96G, and the digestion reaction with the restriction enzymes was performed using *Eco*RV for *HSP60* and *HhaI* for GPI according to the manufacturer’s instructions.

The products were subjected to electrophoresis in a 1.5 % agarose gel and revealed by staining with ethidium bromide.

### PCR of the mitochondrial gene cytochrome oxidase subunit II

For amplification of the gene region comprising a fragment of subunit II of the mitochondrial enzyme Cytochrome oxidase (*COII*) the protocol [30] using TcMit10 and TcMit21 primers was used. Subsequently, 10 μL of the amplified products were digested employing the restriction enzyme *AluI* (Invitrogen, USA, 4 to 12 U/μL) according to the manufacturer’s instructions. The fragments generated were visualized in 6 % polyacrylamide gel stained with silver [26].

### Intergenic spacer miniexon genes of *T. cruzi* (SL-IR)

For amplification of the intergenic region of the miniexon genes the protocol [31] was used employing TcIII and UTCC primers. The analysis of the amplified products was performed in 1.5 % agarose gel stained with ethidium bromide.

### RAPD (Random Amplified Polymorphic DNA)

RAPD analysis following the [32] methodology was used with the objective of verifying the intra-group genetic variability using 10 different primers: A10 - 5’ GTGATCGCAT 3’, A7 - 5’GAAACGGGTG 3’, A15 - 5’ TTCCGAACCC 3’, B15 - 5’GGAGGGTGTT 3’, B19 - 5’ACCCCCGAAG 3’, F13 - 5’ GGCTGCAGAA 3’, F15 - 5’ CCAGTACTCC 3’, N9 - 5’ TGCCGGCTTG 3’, N19 - 5’ GTCCGTACTG 3’, U7 - 5’ CCTGCTCATC 3’.

The RAPD profiles were used to construct a matrix of presence/absence of each band visualized, from which an analysis of similarity was constructed [33] using NTSYSpc software [34]. The relationships between *T. cruzi* strains were estimated using a dendrogram representative of the RAPD data. They were constructed based on DICE coefficient [33] and unweighted pair group analysis (UPGMA) using the Mega 6.04 Beta software. In order to estimate Shannon diversity, cophenetic correlation coefficient, heterozygosity per locus (He) and principle coordinates analysis (PCoA) were performed using GenAlEx 6.5 software. In this case, a genetic distance matrix was constructed. This calculation of pairwise genetic distances for binary data followed the method [35], in which any comparison with the same state yields a value of 0 (both 0 *vs.* 0 comparisons and 1 *vs.* 1 comparisons), while any comparison of different states (0 *vs.* 1 or 1 *vs.* 0) yields a value of 1.

### Ethics

The isolation of the *T. cruzi* samples here characterized was obtained of the patients by blood collection performed after obtaining of a signed consent form approved by the Ethics Committee for Research in Humans from the Centro de Pesquisas René Rachou (CPqRR), FIOCRUZ, Belo Horizonte, MG (Process Number 007/02).

## Results

### Genotyping according to the methodologies *24Sα* rDNA, PCR-RFLP *HSP60* and GPI genes [24]

**Fig. 1:**
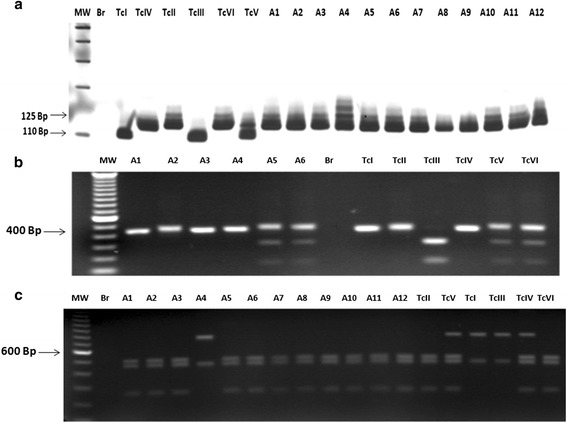
**a** - Profiles obtained by DNA genotyping of *Trypanosoma cruzi* samples isolated from patients of the Jequitinhonha Valley, Minas Gerais, Brazil, resultant from the amplification of the 3' region of the *24Sα* rDNA gene. MW - Molecular weight of 100 bp, Br - negative reaction control, TcI - representative clone of TcI lineage (P209 cl1, 110 bp), TcIV - representative clone TcIV lineage (CANIII, 120 bp), TcII - representative clone of TcII lineage (MAS cl1, 125 bp), TcIII - representative clone of the TcIII lineage (CM-17, 110 bp), TcVI - representative clone of the hybrid lineage TcVI (Tulahuen cl2, 125 bp) and TcV - representative clone of TcV lineage (Bug2148, 110 bp + 125 bp). A1 – sample 229, A2 – sample 452, A3 – sample 748, A4 - sample 798, A5 –sample 205, A6 – sample 1918, A7 –sample 2535, A8 –sample 2491, A9 –sample 264, A10 – sample 376, A11 – sample 646 and A12 – sample 211. **b** - Profiles obtained with DNA of *Trypanosoma cruzi* samples isolated from chagasic patients of the Jequitinhonha Valley, Minas Gerais, Brazil, by the analysis of the *HSP60* gene polymorphism after PCR-RFLP. MW - Molecular weight of 100 bp, A1 – sample 1918, A2 – sample PSF060, A3 – sample 438, A4 – sample 543, A5 – sample 229, A6 – sample 2119. Br – PCR negative control; TcI – TcVI samples profile of the reference clones of the 6 lineages of *T. cruzi* (TcI - P209 cl1, single band of 432 – 462 bp; TcII – MAS cl1, single band of 432 – 462 bp; TcIII – CM-17 double bands; TcIV – CAN III cl1, single band; TcV – Bug 2148, triple bands; TcVI – Tulahuen cl2, triple bands). **c** - Profiles of DNA genotyping of *T. cruzi* samples isolated from chagasic patients of the Jequitinhonha Valley, Minas Gerais, Brazil, by the analysis of *GPI*/*HhaI* gene polymorphism via PCR-RFLP. MW - Molecular weight of 100 bp, BR – PCR negative control, A1 – sample 2014, A2 – sample 646, A3 – sample 453, A4 – sample 2119, A5 – sample 440, A6 – sample 501, A7 – sample 523, A8 – sample 2478, A9 – sample PSF060, A10 – sample 1100, A11 – sample 820, and A12– sample 2535.TcII – (clone TU18 cl1 representative of DTU TcII), TcV – (clone SO3 cl5, DTU TcV), TcI – (clone 92101601P cl1, DTU TcI), TcIII – (Clone X109/2, DTU TcIII), TcVI – (clone Tulahuen, DTU TcVI) and TcIV – (CANIII cl1 DTU TcIV)

According to the criterion [24] 56 out of 63 isolates showed band profiles compatible with TcII DTU: 125 bp (rDNA type I) for *24Sα* rDNA gene products (Fig. 1); a single band for PCR-RFLP *HSP60*/*Eco*RV (Fig. 1 and Table 2) and three bands for PCR-RFLP *GPI*/*HhaI* (Fig. 1 and Table 2) compatible with the reference clone of the TcII DTU (MAScl1). However, with seven isolates it was not possible to reach a consensus following this methodology. Although these seven samples gave the 125 bp fragment for analysis of the polymorphism of D7 *24Sα* rDNA (LSUrDNA), for the other markers there was no correlation with the expected band profiles. Samples 229, 748, 798, 1205, 1337, 2118 and 2119 showed profiles of triple bands for PCR-RFLP of the *HSP60* gene (triple fragments ranging from 100 to 432 bp) characteristic of hybrid lineages or TcV or TcVI (Fig. 1 and Table 2), and double bands, characteristic of the lineages TcI and TcIII for PCR-RFLP of the GPI gene (Fig. 1 and Table 2).

**Table 2:** *Trypanosoma cruzi* genotyping of samples isolated from patients of the Jequitinhonha Valley, MG, Brazil

Molecular typing criteria	*T. cruzi* Samples	
	DTU TcII	DTU TcVI
Lewis et al., (2009)	PSF060, 103, 264, 299, 376, 438, 440, 452, 467, 479, 493, 501, 523, 525, 529, 543, 595, 646, 653, 701, 728, 791, 795, 806, 817, 818, 820, 829, 839, 845, 855, 860, 896, 914, 953, 1100, 1107,1113, 1315, 1442, 1536, 1661, 1662, 1663 1635, 1918, 2014,2336,2337, 2339, 2405, 2408, 2478, 2491, 2495, 2497	?????
D’Ávilla et al., (2009)	PSF060, 103, 264, 299, 376, 438, 440, 452, 467, 479, 493, 501, 523, 525, 529, 543, 595, 646, 653, 701, 728, 791, 795, 806, 817, 818, 820, 829, 839, 845, 855, 860, 896, 914, 953, 1100, 1107, 1113, 1315, 1442, 1536, 1661, 1662, 1663 1635, 1918, 2014, 2336, 2337, 2339, 2405, 2408, 2478, 2491, 2495, 2497	229, 748, 798, 1205, 1337, 2118, 2119
Total (63 samples)	56 samples DTU TcII	7 samples DTU TcVI

### Genotyping according to the methodologies *24Sα* rDNA, PCR-RFLP COII gene and Miniexon SL-IR [16]

**Fig. 2:**
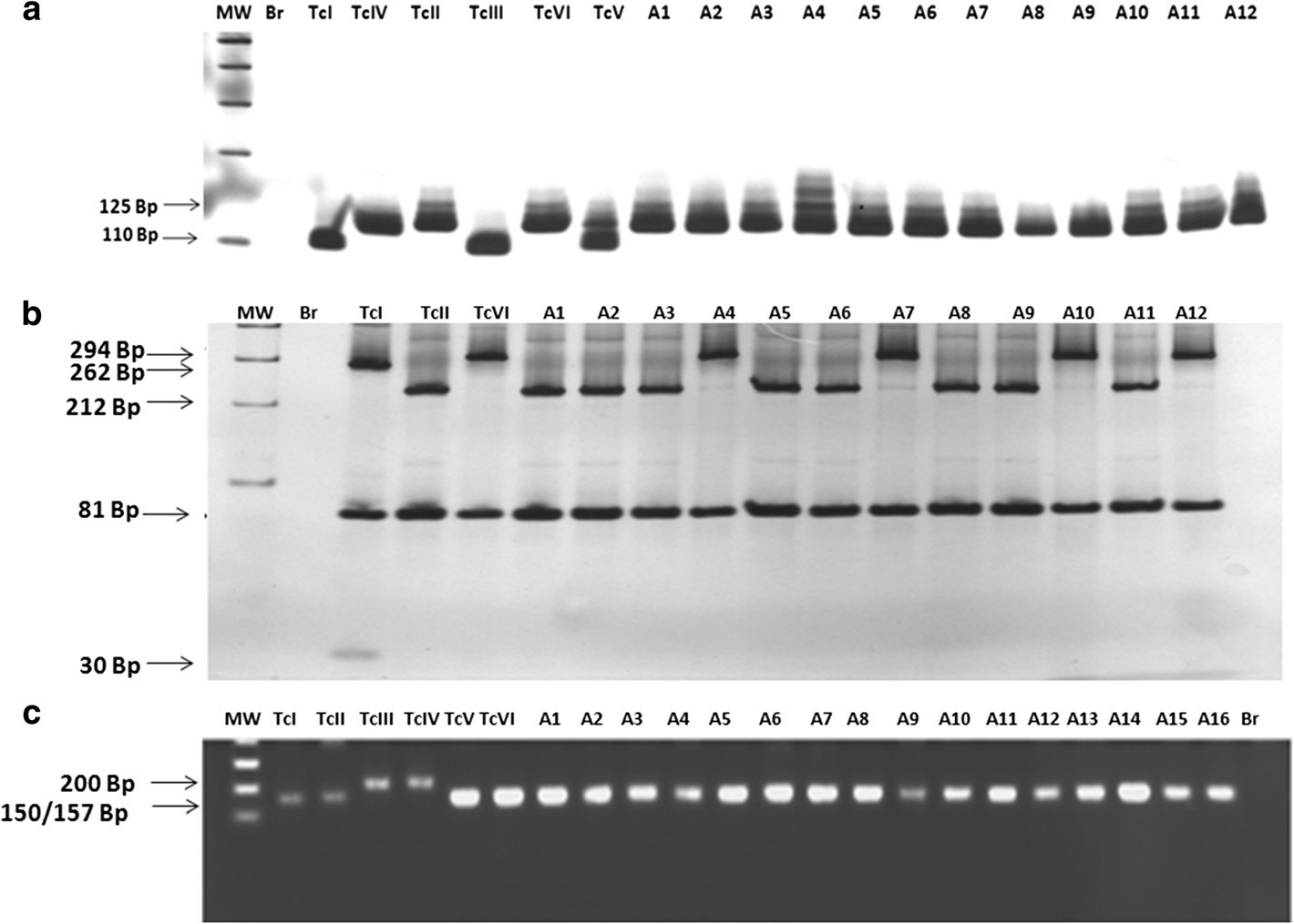
**a** – Profiles obtained by DNA genotyping of *Trypanosoma cruzi* isolated from patients of the Jequitinhonha Valley, Minas Gerais, Brazil, of the region 3' of the *24Sα* rDNA gene. MW - Molecular weight of 100 bp, Br – PCR negative control. TcI – TcI representative clone (P209 cl1 profile of 110 bp), TcIV – representative clone of TcIV (CANIII, 120 bp), TcII - representative clone of TcII(MAS cl1, 125 bp), TcIII – representative clone of the lineage TcIII (CM-17, 110 bp), TcVI – representative of the hybrid lineage TcVI (Tulahuen cl2, 125 bp) and TcV – representative clone of lineage TcV (Bug2148, 110 bp + 125 bp); A1 – sample 229, A2 – sample 452, A3 – sample 748, A4 – sample 798, A5 – sample 1205, A6 – sample 1918, A7 – sample 2535, A8 –sample 2491, A9 – sample 264, A10 – sample 376, A11 – sample 646 and A12 – sample 2118. **b** - Profiles obtained by DNA genotyping of *Trypanosoma cruzi* isolated from patients of the Jequitinhonha Valley, Minas Gerais, Brazil, by analysis of polymorphism of subunit II of the cytochrome oxidase gene (*COII*). MW - Molecular weight of 1 kb, BR – PCR negative control, TcI – profile of bands of clone P209 cl1 (mitochondrial haplotype A - DTU TcI, Freitas et al., 2006), TcII – Tu18 cl2 (haplotype C - DTU TcII), TcVI –M6241 CL6 and C4 – Tulahuen cl2 (mitochondrial haplotype B - DTU TcIII and DTU TcVI); A1 – sample 452, A2 – sample 2535, A3 – sample 376, A4 – sample 229, A5 – 2408 sample, A6 – sample 264, A7 – sample 748, A8 – 1918 sample, A9 – sample 2491, A10 – sample 798, A11 – sample 501, A12 – sample 2118. **c**- Profiles obtained by DNA genotyping of *Trypanosoma cruzi* isolated from patients of the Jequitinhonha Valley, Minas Gerais, Brazil, by analysis of polymorphism of the intergenic spacer of the miniexon. MW - Molecular weight standard 1 kb; C1 – clone Agouti cl1 (DTU TcI), C2 – clone Tu18cl1 (DTU TcII), C3 and C4 – clones X109 2 (DTU TcIII) and 9212210R2 (DTU TcIV), C5 and C6 – clones SO3cl5 and Tula cl2 representatives of DTU (s) TcV and TcVI, respectively; A1 – sample 229, A2 – sample 452, A3 – sample 748, A4 – sample 798, A5 – sample 1205, A6 – sample 1337, A7 – sample 2118, A8 – sample 2119; A9 – sample 376, A10 – sample 2491, A11 – sample 493; A12 – sample 820, A13 – sample 1107; A14 – sample 2478; A15 – sample 1918; A16 – sample 2014, Br: PCR negative control

Due to results obtained above all samples were also analyzed by the protocol proposed by [16]. The fifty-six samples previously identified as TcII by the protocol [24] had the typing confirmed: 125 bp (rDNA type I) for amplified products of *24Sα* rDNA; 212 bp + 81 bp, characteristic of mitochondrial haplotype C lineage and 150 bp −157 bp for amplified products of intergenic spacer of the miniexon (SL-IR) of *T. cruzi*, typical of TcII MAScl1 reference clone. The seven samples with previous controversial identification (229, 748, 798, 1205, 1337, 2118, 2119) showed a double profile of bands of 294 bp and 81 bp (Fig. 2 and Table 2) for PCR-RFLP *COII*, similar to the reference clones of the haplotype B (CM-17 and Tulahuen cl2) or strains belonging to TcIII, TcIV and TcVI lineages. Regarding the amplification of the DNA fragment of the intergenic miniexon spacer (SL-IR) of *T. cruzi*, all samples showed band profiles of approximately 150–157 bp, characteristic of the TcI, TcII, TcV and TcVI lineages [31] (Fig. 2 and Table 2).

Therefore, according to the protocol [16] it was verified that the seven samples with inconclusive results in the protocol proposed by [24] were all classified as belonging to DTUTcVI because they presented a profile of 125 bp fragment for the rDNA gene, approximately 150–157 bp fragment for the intergenic spacer of the miniexon and 294 bp and 81 bp (mitochondrial haplogroup B) which allowed the classification of the *T. cruzi* samples as belonging to TcVI compatible with the Taluhen cl2 reference clone (Table 1) of this *T. cruzi* DTU.

### RAPD analysis

For RAPD analysis only bands showing sharp fragments were selected. After amplification 4731 fragments were detected in the two different DTUs. The size of bands ranged between 100 and 1400 bp.

**Fig. 3:**
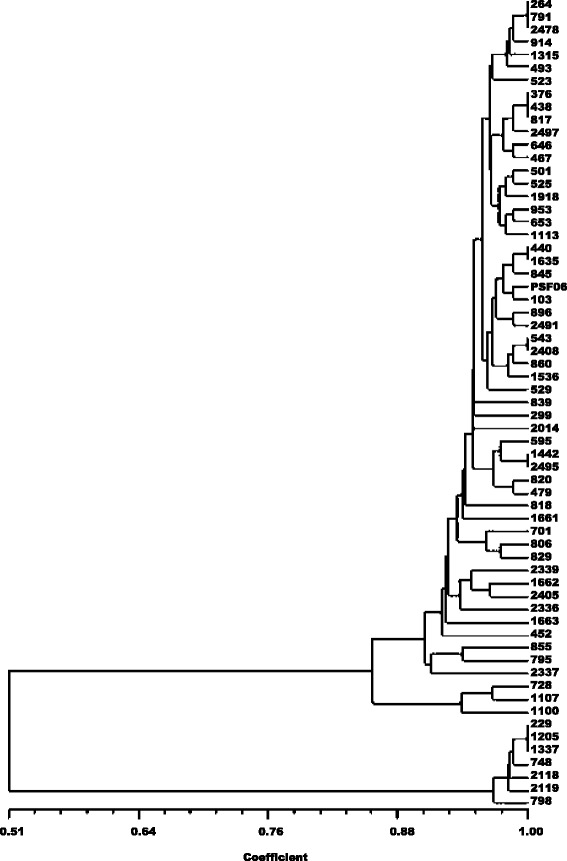
UPGMA (Unweighted Pair-Group Method Analysis) phenogram of all 63 isolates of *Trypanosoma cruzi*. The numbers on the horizontal scale were obtained from the Dice similarity coefficient

**Fig. 4:**
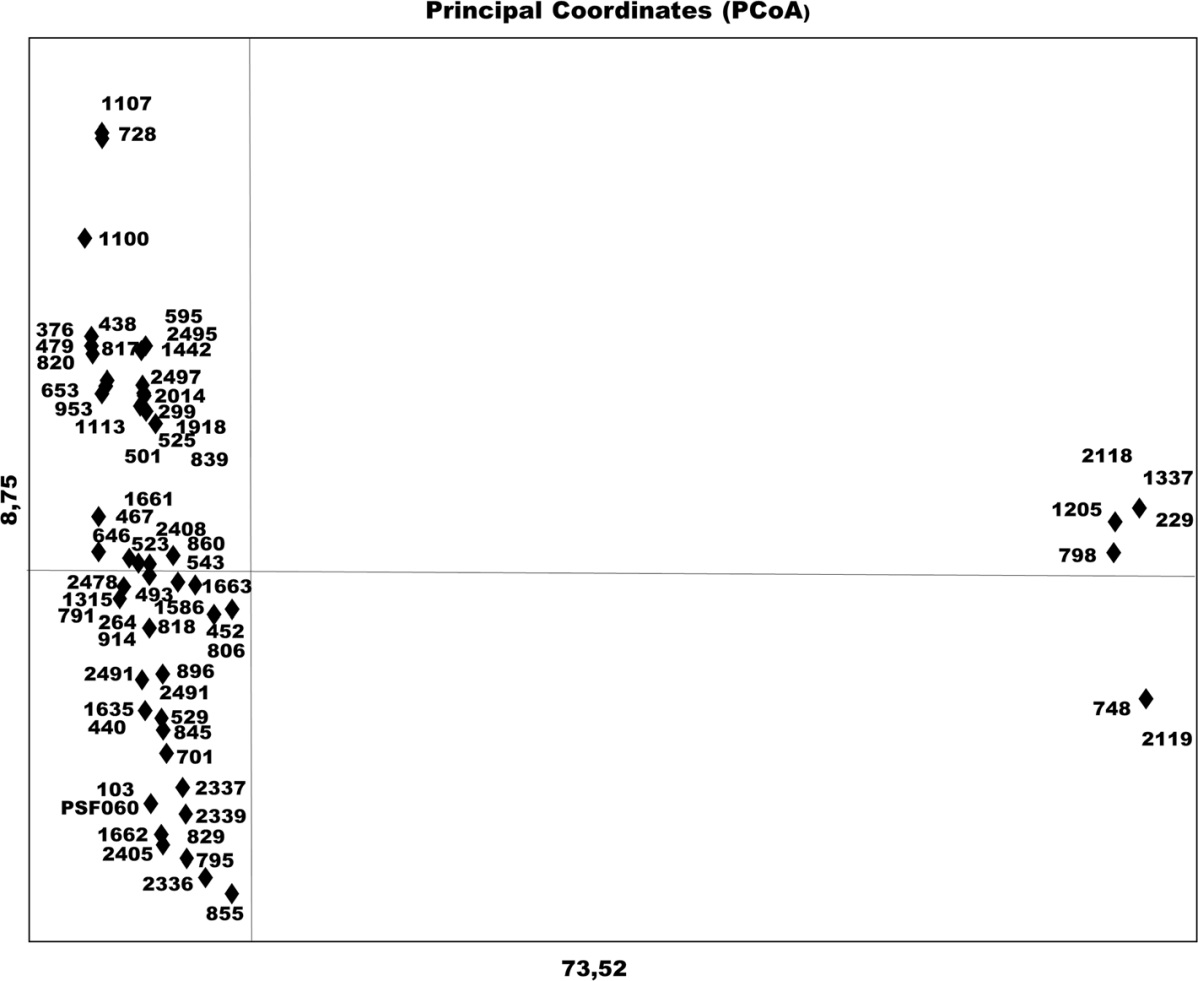
Principal coordinated analysis (PCoA) for the different strains of *Trypanosoma cruzi*

The RAPD profiles were complex, with a total of 81 bands of which only 12 (14.8 %) were shared among all the samples. The proportion of polymorphic loci was 72.84 %. The cophenetic correlation coefficient that checks that a dendrogram faithfully preserves the pairwise distances between the original unmodeled data points gave a coefficient value of 0.989, confirming good representation of the similarity matrices in the form of the dendrogram. The UPGMA dendrogram distinguished the samples into three distinct clusters (Fig. 3). PCoA for the first two axes consolidated 82.27 % of the variation between the components (Fig. 4) and the total formed three groups, as shown in the UPGMA dendrogram. The expected mean heterozygosity per locus (He) was 0.181, indicative of low genetic diversity among samples. Moreover, the Shannon diversity index (0.284), which is a quantitative measure that reflects how many different types there are in a dataset was also considered low. This evaluation has been used to describe the species richness. When this index is closer to 1.0, greater is the species diversity.

## Discussion

*T. cruzi*, the etiologic agent of Chagas disease, is composed of heterogeneous subpopulations circulating in both sylvatic and domestic cycles [36], and this diversity can be detected at morphological [37], biological [3], antigenic [38], epidemiological [4] and genetic levels [36, 39].

Therefore, to better understand the disease it is important to study the molecular epidemiology of this parasite, which naturally is related with the characteristics mentioned. So, the present study was developed with the purpose of identification of the genetic lineages of *T. cruzi* samples isolated from chronic chagasic patients of the Jequitinhonha Valley, MG, considered an area of intense transmission of Chagas disease in the 1980s [40] and (ii) to evaluate the molecular descriptive epidemiology of *T. cruzi* within this population.

For molecular typing it was firstly used the protocol proposed by [24]. Considering the 63 samples evaluated, a total of 56 *T. cruzi* samples showed a profile consistent with TcII as previously demonstrated in patients of Virgem da Lapa municipality, located only 28 km from Berilo and 48 km from José Gonçalves de Minas, where the isolates were analyzed by isoenzyme profiles [41]. The first work that used the criteria proposed by [24] for *T. cruzi* genotyping was the study [42] as mentioned by [4]. They identified 18 isolates from domestic cats and vectors in an endemic region of Chagas disease in Bahia, all belonging to the TcII group. Our results corroborate the obtained in this study, indicating that this method is efficient in identifying TcII DTU. However, seven samples (229, 748, 798, 1205, 1337, 2118 and 2119) revealed different patterns or combinations of bands to those expected for any DTU. The results obtained by protocol [24] already indicated genetic variation in the profiles of some clones used as reference in their work. The clone Saimiri3 cl1, for example, showed a profile of TcIV in RAPD analysis. However, the triple assay showed inconclusive identification closer to the profile of TcII than any other genetic group. The most important issue that may hinder the identification of the isolates is the occurrence of mixed infections or multiconality already documented in vectors, several reservoirs and human [38], but fortunately not identified in our samples since the profiles of bands detected where exactly the same verified in clones of reference for each *T. cruzi* DTU and because the pattern of bands for each one is intra-DTU excludent [6]. Additionally, when a mixture of TcIII and TcII clones occurred in [43] these authors found similar profiles to TcV and TcVI, which could result in erroneous identification or characterization of the isolates, since the samples studied by them were not cloned previously to clarify this problem.

Herein, the results showed that more genetic variability may be observed when more samples from distinct geographic areas are typed using this protocol. For our seven samples that showed different patterns of bands for the expected by the protocol [24] a process may be occurring, such as nucleotides deletion or insertion of the GPI gene sequence, which prevented the enzyme to cut the DNA sequence at the specific site. On the other hand, [44, 45] publications do not have problems with the use of GPI gene as a marker, however employing different protocols. The isoenzyme analysis of these isolates for GPI also showed a profile of bands distinct from that expected for the TcVI stocks (data not shown). Our analysis of GPI showed profiles corresponding to those expected for TcIV and TcIII DTUs, which could suggest a more extensive variation in the gene, but for others (G6PD and IDH) the profile was compatible with TcVI (data not shown). Regarding the use of the *HSP60* gene as a marker, there are not many publications that deal with this methodology, except the works [27] and [28]. In this study we did not observe differences between the representative samples of the same lineage for these two genes using the PCR-RFLP. However, it is important to note those authors used clones of references previously genotyped by others markers, as well as different restriction enzymes to cut the HSP60 and GPI gene fragments, which could explain the absence of differences observed compared to the present study that genotyped new samples isolated from humans.

To solve the typing of these seven samples the protocol provided by [16] was also employed in the characterization of all samples. According to [31] when incongruity occurs in the identification of *T. cruzi* isolates, with probable presence of a hybrid profile, the use of PCR-RFLP of the *COII* gene described by [30] is important for the characterization of the isolate to guarantee the right profile identification because after cutting the PCR products with the AluI enzyme, samples associated with the mitochondrial haplogroup B profile can be considered as belonging to the DTUs TcIII, TcIV, TcV or TcVI. However, employing this methodology combined with the evaluation of the polymorphism of the 3' rDNA gene region and of the intergenic region of the miniexon gene of *T. cruzi* (SL-IR) also suggested a quick, cheap and effective solution for identifying strains of *T. cruzi* as suggested by [6] and [4]. Here, seven isolates from chronic chagasic patients (229, 748, 798, 1205, 1337, 2118 and 2119) after *COII*/*Alu* RFLP showed profiles of representative clones of mitochondrial haplotype B (294 bp + 81 bp) and that the DTU observed may correspond to hybrids DTUs V and VI. The SL-IR identified the group with a band of 200 bp, characteristic of the lineages TcIII and TcIV. Thus, all samples analyzed in this study amplified by this methodology showed a fragment of approximately 150–157 bp, excluding the possibility of the isolates being identified as TcIII or TcIV, which confirms that these samples are actually hybrids belonging to DTU TcVI, due also to the results obtained in the rDNA analysis. The other 56 samples were confirmed as TcII genotype, including the only sample of José Gonçalves de Minas, which corroborated the classification of [24].

Thus, the results of this study are in agreement with several publications that claim that TcII strains are more associated with human infection with geographical distribution between the North and mainly in the South of Brazil [12, 15, 16, 46, 47]. In a recent review encompassing the ecoepidemiology of different DTUs of *T. cruzi,*[4] reaffirm this distribution and that this DTU was detected both in the domestic and sylvatic cycles of Chagas disease [48, 49] and appears to be closely related to the cardiac and digestive clinical manifestations (megacolon and megaesophagus) in human infection [12, 16, 30] Predominance of 91.7 % of TcII in Brazil was demonstrated in human cases in the state of Rio Grande do Norte [50] similar to that observed in the Jequitinhonha Valley, MG. These authors also verified presence of TcII in domestic reservoirs. On the other hand, the presence of hybrid groups represented by the DTUs TcV and TcVI is usually associated with vectors and humans in the domestic cycle of infection and rarely recorded in the sylvatic cycle in various geographical regions of Latin America [51, 52]. Frequently, TcVI presents low prevalence in the invertebrate host than in human cases and some mammal reservoirs [10, 52–54]. Several publications have also cited TcVI as an important lineage involved in human infections in the Chaco region and neighboring countries such as Bolivia, Chile, northern Argentina and southern Brazil [55–57]. The existence of TcVI in humans in Brazil was recently confirmed by [58] when they characterized 11 strains isolated from patients involved with oral transmission of *T. cruzi* in the state of Santa Catarina. More recently, the study [8] in Colombia demonstrated the presence and association of TcI, TcII, TcIII and TcIV DTUs, mainly TcI and not TcII as generally demonstrated by different authors in distinct regions such as Venezuela and Bolivia [7, 9, 10, 15, 30, 46]. To evaluate the variability within these genetic groups RAPD analysis was used on all samples here characterized. This analysis clearly demonstrated the subdivision of this species into two genetic lineages and corroborated the results obtained by the genotyping of all isolates, grouping them equally with the reference clones of the DTUs II and VI as previously demonstrated. In addition, a great similarity between the profiles of bands presented by the samples of the same lineage was observed using the different primers, showing only discrete variability among them. The studies [5, 59] showed a tendency for decreasing genetic variability of the isolates when comparing parasites isolated from patients in the acute phase with patients in the chronic phase of the infection using RAPD and microsatellite methodologies. These results are consistent with the idea that clones of *T. cruzi* are able to establish stronger infections in certain hosts and such host-parasite relationships could work as filters for some sub-populations of the parasite [5, 49, 60]. Maybe the prolonged infection in these patients may have provided a selection of the parasites better adapted to the Berilo population or the ecoepidemiological conditions of the study area. This hypothesis justifies the similarity between the isolates grouped in a particular branch of the phenogram.

Phenomena of *T. cruzi* hybridization and ecogeographical distribution of the parasite were also described by other authors referent to TcI in Bolivia, employing loci variability by microsatelites and mitochondrial DNA [7] and between TcI with TcIII and TcIV, respectively [61] using MLST and SNP as molecular markers. However, the study of parasites isolated from reservoirs and vectors of the same region would be necessary to better understand the eco-epidemiology of *T. cruzi* in the region studied.

## Conclusions

The results obtained in this study agree with data in the literature and demonstrate the predominance of the lineage TcII in human Chagas disease in more one endemic region of Brazil, as well as its association with genotype TcVI, now firstly described in human infection in the Jequitinhonha Valley, MG, Brazil, which may have been masked in the pioneer classification here used due to the limitations of the markers used which probably erroneously identified those samples as possibly TcII.

The RAPD analysis showed low variability of the genetic profiles between *T. cruzi* samples of the same lineage.

The results still suggest variation among samples from different geographic regions, and more, that the correct identification of *T. cruzi* DTUs should be made with caution and based on a larger number of markers, mainly when hybrid samples are present.

## Abbreviations

DTU: Discrete Typing Unit
TcI: *Trypanosoma cruzi* I DTU
TcII: *Trypanosoma cruzi* II DTU
TcIII: *Trypanosoma cruzi* III DTU
TcIV: *Trypanosoma cruzi* IV DTU
TcV: *Trypanosoma cruzi* V DTU
TcVI: *Trypanosoma cruzi* VI DTU
MG: Minas Gerais state, Brazil
RJ: Rio de Janeiro state, Brazil
RAPD: Randomly amplified Polymorphic DNA
IRD: Institute de la Recherche pour le Dévelopment
LIT: Liver Infusion Tryptose
PBS: Phophate Buffer Solution
Rpm: Rotation per minute
DNA: Desoxiribonucleotide
RNA: Ribonucleotide
PCR: Polymerase chain reaction
RFLP: Restriction fragment length polymorphism
HSP60: Heat shock protein
GPI: Glucose phosphate isomerase
*AluI*: Restriction endonuclease from *Arthrobacter luteus*
UPGMA: Unweighted pair group analysis
bp: Base pair
MW: Molecular weight
